# Comparison of the Immunogenic Properties of *Lactiplantibacillus plantarum* Carrying the Mycobacterial Ag85B-ESAT-6 Antigen at Various Cellular Localizations

**DOI:** 10.3389/fmicb.2022.900922

**Published:** 2022-06-03

**Authors:** Kamilla Wiull, Preben Boysen, Katarzyna Kuczkowska, Lars Fredrik Moen, Harald Carlsen, Vincent G. H. Eijsink, Geir Mathiesen

**Affiliations:** ^1^Faculty of Chemistry, Biotechnology and Food Science, NMBU - Norwegian University of Life Sciences, Ås, Norway; ^2^Faculty of Veterinary Medicine, NMBU - Norwegian University of Life Sciences, Ås, Norway

**Keywords:** mucosal vaccine platform, *Lactiplantibacillus plantarum*, delivery vector, tuberculosis, lactic acid bacteria, surface display

## Abstract

The bacille Calmette-Guèrin (BCG) vaccine has been used for a century; nonetheless, tuberculosis (TB) remains one of the deadliest diseases in the world. Thus, new approaches to developing a new, more efficient vaccine are desirable. Mucosal vaccines are of particular interest, considering that *Mycobacterium tuberculosis* first enters the body through the mucosal membranes. We have previously demonstrated the immunogenicity of a recombinant *Lactiplantibacillus plantarum* delivery vector with TB hybrid antigen Ag85B-ESAT-6 anchored to the cell membrane. The goal of the present study was to analyze the impact of antigen localization in the immune response. Thus, we assessed two novel vaccine candidates, with the TB antigen either non-covalently anchored to the cell wall (LysMAgE6) or located intracellularly (CytAgE6). In addition, we compared two expression systems, using an inducible (LipoAgE6) or a constitutive promoter (*c*LipoAgE6) for expression of covalently anchored antigen to the cell membrane. Following administration to mice, antigen-specific CD4^+^ T-cell proliferation and IFN-γ and IL-17A secretion were analyzed for lung cell and splenocyte populations. Generally, the immune response in lung cells was stronger compared to splenocytes. The analyses showed that the type of expression system did not significantly affect the immunogenicity, while various antigen localizations resulted in markedly different responses. The immune response was considerably stronger for the surface-displaying candidate strains compared to the candidate with an intracellular antigen. These findings emphasize the significance of antigen exposure and further support the potential of *L. plantarum* as a mucosal vaccine delivery vehicle in the fight against TB.

## Introduction

*Mycobacterium tuberculosis* is the causative agent of tuberculosis (TB) and ranked by the World Health Organization (WHO) as the number one cause of death from a single infectious agent. An estimated quarter of the world’s population is infected with *M. tuberculosis,* despite the introduction of the bacille Calmette-Guérin (BCG) vaccine a century ago, which is one of the most widely used vaccines. The BCG vaccine provides good protection against severe forms of TB in children, but protection against pulmonary TB and overall adult protection are highly variable (Mangtani et al., 2014; Li et al., 2020). Transmissible cases of TB disease occur more often in adults than in children (Pai et al., 2016), urging the need for alternative vaccines, which is further strengthened by the increased prevalence of drug-resistant *M. tuberculosis* (Pai et al., 2016). There are currently two vaccine candidates in the third and last phase of clinical trials, while 12 vaccines are in phase I or II. The late phase candidates are predominantly mycobacterial-based or viral delivery vectors (World Health Organization, 2020).

To date, no non-mycobacterial vectored TB vaccines are in clinical trials, despite extensive research (Pereira et al., 2017; Jiang et al., 2020). The use of bacterial strains with attenuated pathogenicity is an interesting approach as these vectors may act as adjuvants and be particularly efficient at inducing cellular immune responses (Pollard and Bijker, 2021). However, attenuated pathogens face limitations due to the risk of being reverted back to its pathogenicity and pre-existing immunity in the host (Lund and Randall, 2021; Pollard and Bijker, 2021). The risk of reversion is a particular disadvantage for TB vaccines, considering more than 8% of people with TB are co-infected with Human Immunodeficiency Virus (HIV; World Health Organization, 2020). Hence, utilization of non-pathogenic vectors, such as lactic acid bacteria (LAB), is therefore a safer and more viable future strategy (LeCureux and Dean, 2018).

Many LAB are a natural part of the human microbiome with a generally regarded as safe (GRAS) status (Szatraj et al., 2017). Furthermore, many species of LABs are easy to cultivate and store and several expression systems have been developed to produce heterologous proteins (Wyszyńska et al., 2015). In recent years there has been an increasing interest in exploitation of bacteria of the *Lactobacillales* order as potential vaccine vectors (LeCureux and Dean, 2018; Mustafa et al., 2018; Wang et al., 2020). One promising candidate is *Lactiplantibacillus plantarum,* being one of the most researched species of the *Lactobacillales* order. *L. plantarum* is known to have beneficial immunogenic properties and may contribute to activation of both innate and adaptive immunity (Bloksma et al., 1979; de Vos et al., 2017).

The use of *L. plantarum* as a vaccine vector is particularly interesting in the development of mucosal vaccines (LeCureux and Dean, 2018). Though parenteral vaccination effectively induces a systemic immune response, mucosal immunity is best acquired by direct administration of the vaccine to the mucosa, which can be achieved by oral or intranasal administration (Holmgren and Czerkinsky, 2005; Neutra and Kozlowski, 2006; Lycke, 2012; Lund and Randall, 2021). *M. tuberculosis* enters the body through mucosal surfaces before the infection is established in the lung (Pai et al., 2016). Therefore, it would be advantageous for a new TB vaccine to generate mucosal immunity and activate immune cells of the lung (Thomas and McShane, 2015).

The accessibility (i.e., exposure) of the vectored antigen likely affects the interaction between the antigen and the targeted immune cells (Lee et al., 2000; Bermúdez-Humarán et al., 2004). At the same time, the antigen should be sufficiently protected from extracellular degradation. Different localization and exposure may be obtained by varying the presence of a signal peptide that directs the antigen to the outside of the cell and by using different anchors that may covalently anchor the antigen to the cell membrane or the cell wall, or that may promote non-covalent anchoring to the cell wall through so-called LysM domains (Michon et al., 2016).

A recent study showed that intranasal immunization of mice with recombinant *L. plantarum* displaying a membrane-bound fusion antigen derived from *M. tuberculosis*, Ag85B-ESAT-6, yielded a higher number of proliferating peripheral blood mononuclear cells (PBMC) compared to bacteria where the antigen was covalently anchored to the cell wall (Kuczkowska et al., 2017a). *In vitro* analyses have suggested that LysM anchoring to the cell wall leads to more exposed proteins compared to proteins that are covalently anchored through a LPxTG motif (Mathiesen et al., 2020).

**Figure 1:**
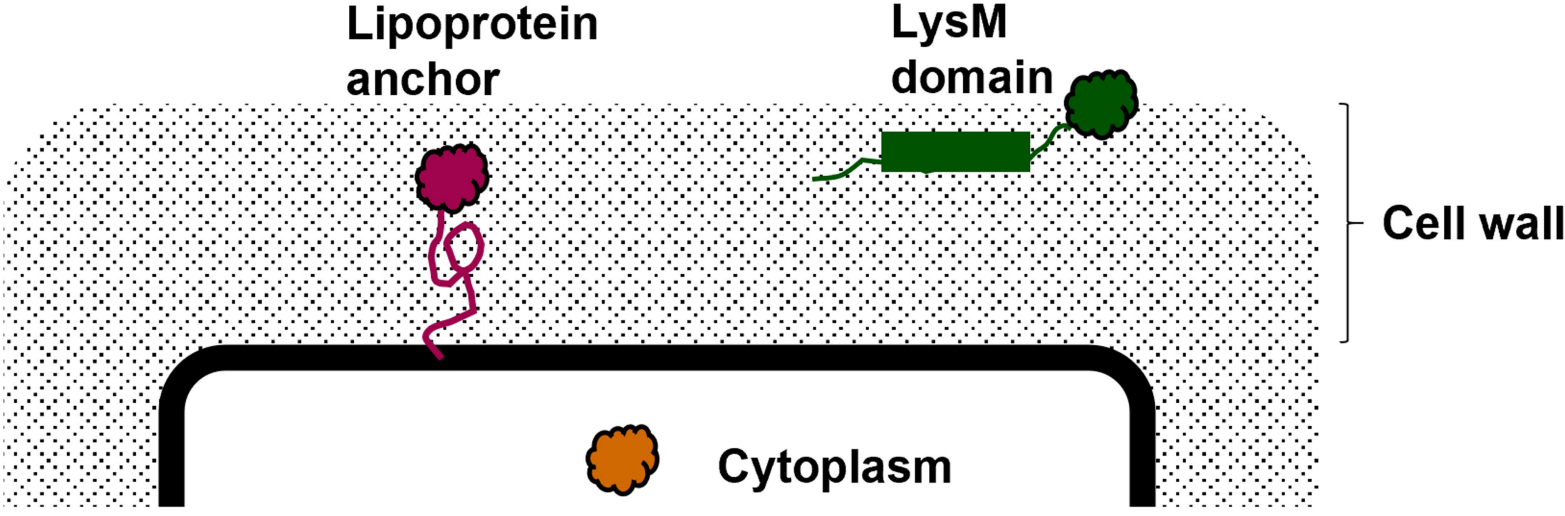
A schematic presentation of the vaccine candidates with different levels of antigen exposure. The picture illustrates cytoplasmic localization of the antigen (orange), covalent anchoring of the antigen to the cell membrane through a lipoprotein (magenta) and non-covalent anchoring to the peptidoglycan cell wall through a LysM domain (green).

In the present study, we have used the pSIP expression system (Sørvig et al., 2003, 2005) to generate strains of *L. plantarum* WCFS1 that contain the *M. tuberculosis* fusion antigen Ag85B-ESAT-6 (AgE6) at three subcellular locations: cytoplasm (CytAgE6), covalently anchored to the cell membrane (LipoAgE6/*c*LipoAgE6) or non-covalently anchored to the cell wall through a LysM domain (LysMAgE6). This should create three levels of exposure, varying from not at all (cytoplasmic) to buried in the cell wall (membrane anchor) to maximally exposed (LysM anchor; Figure 1). Since the nature of the expression system may also play a role (Bermúdez-Humarán et al., 2004), two variants of the strain producing AgE6 that is covalently anchored to the cell membrane (lipoprotein-anchored) were generated, one with the inducible pSIP expression system (LipoAgE6) and one with constitutive expression (*c*LipoAgE6). The vaccine candidate strains were characterized in terms of bacterial growth, as well as production and surface display of the antigen. Furthermore, the candidates were tested *in vivo* in a mouse model for their ability to induce a humoral immune response in the mucosa and a cellular immune response in the lung and spleen. Immune responses were assessed by measuring production of antigen-specific IgA in lung washes, as well as CD4^+^ T-cell proliferation, IFN-γ, and IL-17A in lung cells and splenocytes. Specific immune responses were detected for all recombinant *L. plantarum* strains, and we show how the magnitude of these responses depends on antigen localization.

## Materials and Methods

### Bacterial Strains, Plasmids, and Growth Conditions

The bacterial strains and plasmids used in this study are listed in Table 1. *Escherichia coli* was cultivated in BHI medium (Oxoid Ltd., Basingstoke, United Kingdom) at 37°C with agitation. Erythromycin was added to a final concentration of 200 μg/ml. *L. plantarum* was cultured in MRS broth (Oxoid Ltd) without agitation at 37°C. Erythromycin was added to a final concentration of 10 μg/ml. For plates, liquid BHI and MRS medium were solidified by adding 1.5% (w/v) agar.

**Table 1:** Plasmids and strains used in this study.

**Plasmid**	**Description**	**References**
pEV	Ery^r^; 256_rep_; pSIP401 derivative; control plasmid (“empty vector”);	Fredriksen et al., 2012
pLp_cyt:AgE6-DC	Ery^r^; 256_rep_; pSIP401 derivative; containing the inducible P*_sppA_* fused to a gene construct encoding Ag85B-ESAT6 followed by a dendritic cell binding sequence (DC).	This study
pLp_1261AgE6-DC	Ery^r^; 256_rep_; pSIP401 derivative; containing the inducible P*_sppA_* fused to a gene construct encoding the lipoprotein anchor sequence from the gene *lp_1261* followed by the sequence encoding Ag85B-ESAT-6 and the DC sequence.	Kuczkowska et al., 2017a
pSlpA_1261ManB	Ery^r^; 256_rep_; pSIP401 derivative; containing the constitutive promoter P_slpA_ from *Lactobacillus acidophilus* ATCC4356 fused to the *manB* gene.	Nguyen et al., 2019
pLp_SlpA-1261AgE6-DC	Ery^r^; 256_rep_; pSIP401 derivative; containing P*_slpA_* fused to a gene construct encoding the lipoprotein anchor sequence *lp_1261* followed by the sequence encoding Ag85B-ESAT-6 and the DC sequence.	This study
pLp_3014AgE6-DC	Ery^r^; 256_rep_; pSIP401 derivative; containing the inducible P*_sppA_* fused to a gene construct encoding the N-terminal signal peptide and LysM anchor derived from the gene *lp_3014* followed by the sequence encoding Ag85B-ESAT-6 and the DC sequence.	This study
pLp_3014Inv	Ery^r^; 256_rep_; pSIP401 derivative; encoding Invasin fused to an N-terminal signal peptide and LysM anchor derived from *lp_3014.*	Fredriksen et al., 2012
mcherry-pBAD	Amp^r^; encoding red fluorescent protein mCherry.	Addgene (Watertown, MA)
pSIP403_mCherry	Ery^r^; 256_rep_; pSIP403 derivative; encoding red fluorescent protein mCherry under control of the inducible P*_sppA_*.	This study
pSIP403_SlpA_mCherry	Ery^r^; 256_rep_; pSIP403 derivative; encoding red fluorescent protein mCherry under control of the constitutive P_slpA_.	This study
**Strains**	**Description**	**References**
*Escherichia coli* TOP10	Subcloning strain	Invitrogen
*Lactiplantibacillus plantarum* WCFS1	Host strain	Kleerebezem et al., 2003
pEV	*L. plantarum* harboring pEV	Fredriksen et al., 2012
LipoAgE6	*L. plantarum* harboring pLp_1261AgE6-DC	Kuczkowska et al., 2017a
*c*LipoAgE6	*L. plantarum* harboring pLp_SlpA-1261AgE6-DC	This study
LysMAgE6	*L. plantarum* harboring pLp_3014AgE6-DC	This study
CytAgE6	*L. plantarum* harboring pLp_cyt:AgE6-DC	This study
P*_sppA_*-mCherry	*L. plantarum* harboring pSIP403_mCherry	This study
P_s*lpA*_-mCherry	*L. plantarum* harboring pSIP403_SlpA_mCherry	This study

### Plasmid Construction and DNA Manipulation

The construction of plasmids pEV and pLp_1261AgE6-DC has been described previously (Table 1; Fredriksen et al., 2012; Kuczkowska et al., 2017a). All antigen-carrying plasmids contained the Ag85B-ESAT-6 antigen translationally fused to a dendritic cell binding (DC) peptide (Curiel et al., 2004), this protein will hereafter be referred to as AgE6.To construct pLp_3014AgE6-DC, the DC-tagged AgE6 hybrid antigen was amplified using pLp_1261AgE6-DC as template with the primer pair Ag85BFus3014F/Ag85DC-R (Table 2). Both the AgE6 PCR-fragment and the pLp_3014Inv vector were digested with *Sal*I/*Eco*RI before ligation and transformation into *E. coli*.

**Table 2:** Primers used in this study.

Primer	Sequence (5′ ➔ 3′)^a^	Description
NdeISIP_F	GAGTATGATT*CATATG*TTTAGTCGTCCAGGTTTGC	Forward primer for amplification of AgE6 from pLp_1261AgE6-DC Contains an *Nde*I restriction site.
AgE6-DC_HindIII-R	TGTAATTTG*AAGCTT*TTATGGCCGTTGTGGCGTA	Reverse primer for amplification of AgE6 from pLp_1261AgE6-DC. Contains a *Hind*III restriction site.
Ag85Fus3014F	CAACGAGTTCAACT*GTCGAC*TTTAGTCGTCCAGGTT	Forward primer for amplification of AgE6 from Lp_1261AgE6-DC. Contains a *Sal*I restriction site.
Ag85DC-R	GCCAAGCTTC*GAATTC*TTATGGCCGTTGTGGCGT	Reverse primer for amplification of AgE6 from Lp_1261AgE6-DC. Contains an *Eco*RI restriction site.
SekF	GGCTTTTATAATATGAGATAATGCCGAC	Forward primer used for amplification of P*_slpA_* from pSlpA_1261ManB.
SlpA_1261R	GTTTTGAAATT*CATATG*GTCTTTTCCTCCTTGAAATATAA	Reverse primer for amplification of P*_slpA_* from pSlpA_1261ManB. Contains an *Nde*I restriction site.
Cherry_F	GGAGTATGATT*CATATG*AGCAAAGGAGAAGAAGATAAC	Forward primer for amplification of mCherry from mcherry-pBAD. Contains an *Nde*I restriction site
Cherry_R	CTGTAATTTG*AAGCTT*TTATTTGTAAAGCTCATCCATTCCGC	Reverse primer for amplification of mCherry from mcherry-pBAD. Contains a *Hind*III restriction site.

a Italics indicate restriction sites.

The plasmid used for cytoplasmic production of AgE6 was constructed by amplifying AgE6, with pLp_1261AgE6-DC as template, using the primer pair NdeISIP_F/AgE6-DC_HindIII_R (Table 2). Both the PCR product and pLp_1261AgE6-DC were digested with *Nde*I/*Hin*dIII and subsequently ligated and transformed into *E. coli* yielding pLp_cyt:AgE6-DC. The plasmid used for constitutive expression of the antigen was constructed by amplification of P*_slpA_* (Boot et al., 1996) using the primer pair SekF/SlpA_1261R and pSlpA_1261ManB (Table 2; Nguyen et al., 2019) as template. The SlpA amplicon and the pLp1261AgE6-DC vector were both digested with *Bgl*II/*Nde*I, ligated, and transformed to *E. coli*, yielding pLp_SlpA-1261AgE6-DC. Transformation of the plasmids pLp_1261AgE6-DC, pLp_SlpA-1261AgE6-DC, pLp_3014AgE6-DC, and pLp_cyt:AgE6-DC to *L. plantarum* yielded the vaccine candidate strains LipoAgE6, *c*LipoAgE6, LysMAgE6, and CytAgE6, respectively (Table 1).

To construct pSIP403_mCherry, the mCherry gene was amplified from the mcherry-pBAD plasmid with InFusion primers Cherry_F/Cherry_R (Table 2). The vector, pLp_1261AgE6-DC, was digested with *Nde*I and *Hin*dIII. Subsequently, mCherry was cloned into the digested vector following the InFusion (Takara Bio, Kusatu, Japan) protocol, and transformed to *E. coli*. To construct pSIP403_SlpA_mCherry, pSIP403_mCherry and pLp_SlpA-1261AgE6-DC were digested with *Nde*I/*Hin*dIII, ligated using ElectroLigase (New England BioLabs, MA, United States), followed by transformation directly into *L. plantarum*.

All PCR-derived plasmids were sequenced before transformation to *L. plantarum*. *L. plantarum* was made electrocompetent and transformed as previously described (Aukrust and Blom, 1992).

### Production of Antigen and Treatment of Bacteria

Overnight cultures were diluted in prewarmed MRS to an OD_600_ of 0.15. Antigen expression using an inducible promoter was obtained by addition of 25 ng/ml SppIP (Eijsink et al., 1996; Caslo ApS, Lyngby, Denmark) after approximately 2 h of growth, when the cultures reached an OD_600_ of 0.30 (Mathiesen et al., 2022). The induced strains were harvested 3 h after induction by centrifugation (5,000 × g for 10 min). The strain *c*LipoAgE6 with constitutive expression of the antigen was diluted in prewarmed MRS to an OD_600_ of 0.15 and incubated for 5 h at 37°C before harvesting by centrifugation (5,000 × g for 10 min). Harvested cells were stored at −20°C prior to preparation for western blot analysis or at 4°C (1 day) prior to preparation for flow cytometry analysis.

### Analysis of mCherry Fluorescence

Overnight cultures of P*_sppA_*-mCherry and P*_slpA_*-mCherry were diluted in prewarmed MRS to an OD_600_ of 0.15. The strain with mCherry expression controlled by P*_sppA_* was induced by addition of 25 ng/ml SppIP after ~2 h of growth, when the cultures reached an OD_600_ of 0.30. Another culture of this strain was not induced, but otherwise treated equally, to provide a negative control. The strains grew at an equal rate (data not shown), and both the inducible and constitutive mCherry strains were analyzed during the same time period (i.e., the first 3 h after induction of P*_sppA_-*mCherry). For analysis, 200 μl of the cultures were transferred to a black 96-well plate for measurement of fluorescence (Excitation 587 nm, Emission 620 nm) using a Varioskan Lux Reader set at 37°C (Thermo Fisher Scientific). The fluorescent signal was measured after 3 h.

### Western Blot

Western blot analysis was performed to verify the production of AgE6 in the recombinant *L. plantarum* strains (Table 1). The harvested bacterial cells were resuspended in 1 ml PBS and lysed using a FastPrep FP120 Cell Disrupter (MP Biomedicals, Santa Ana, CA) with three cycles of shaking at 6.5 m/s for 45 s. Subsequently, the samples were centrifuged at 16,000 g for 1 min at 4°C to remove cell debris. Samples of the resulting extracts were mixed with NuPAGE^™^ Sample Reducing Agent (10X; Thermo Fisher Scientific, Waltham, MA) and NuPAGE^™^ LDS Sample Buffer (4X; Thermo Fisher Scientific), and boiled for 10 min prior to loading onto the SDS-PAGE gel. The volume of the cell-free protein extracts applied to the SDS-PAGE gel was normalized based on the OD_600_ at the time of harvesting. After electrophoresis, proteins were transferred to a PVDF membrane using the eBlot L1 Fast Wet Transfer system (Genscript). Following transfer, the SNAP id 2.0 kit (Sigma-Aldrich, Saint-Louis, MO) was used for antibody hybridization to AgE6 according to the manufacturer’s protocol. The mouse monoclonal anti-ESAT-6 (ab26246, Abcam, Cambridge, United Kingdom) primary antibody was diluted 1:5,000, the polyclonal HRP-conjugated anti-mouse IgG (Sigma-Aldrich) secondary antibody was diluted 1:15,000. The proteins were visualized using the SuperSignal West Pico PLUS Chemiluminescent substrate (Thermo Fisher Scientific) and the signals were imaged with an Azure c400 system (Azure biosystems, Dublin, CA).

### Detection of Surface-Displayed Proteins by Flow Cytometry and Fluorescence Microscopy

Bacterial cultures were grown and induced as described above. Flow cytometry analysis was performed to verify surface display of AgE6 in *L. plantarum*. Cells harvested from approximately 500 μl culture were washed once with PBS. The pellet was then resuspended in a 1:250 dilution of the primary antibody anti-ESAT-6 (Abcam) in PBS/2% (w/v) BSA followed by incubation for 30 min at room temperature. Subsequently, the bacteria were washed two times with 600 μl PBS/2% BSA. After the second washing step, the pellet was resuspended in a 1:170 dilution of FITC-conjugated anti-mouse IgG secondary antibody (Sigma-Aldrich) in PBS/2% BSA followed by incubation for 30 min at room temperature, protected from light. The cells were then washed three times with PBS/2% BSA before subsequent analysis using a MACSQuant analyzer (Miltenyi Biotec GmbH, Bergisch Gladbach, Germany). The data were analyzed using FlowJo software. Bacterial samples used for fluorescence microscopy were prepared in the same manner and analyzed with a Zeiss LSM 700 Confocal Microscope using Zen software. Image analysis was performed using the ImageJ plugin MicrobeJ (Ducret et al., 2016). The phase contrast images were used for determination of the shape and size of the bacterial cell, and the FITC signal intensities were extracted from the corresponding fluorescent images.

### Animals and Housing Conditions

The animal experiment described in the present study was approved by the Norwegian Animal Research Authority (FOTS ID 23585). 6- to 8-week-old female C57BL/6JRj mice were purchased from Janvier (Genest St. Isle, France). The mice were housed in pathogen-free, individually ventilated cages (Innovive Inc., San Diego, CA) with food and water *ad libitum*, under standard housing conditions; 12-h light/dark cycle, 23°C–25°C, and 45%–50% relative humidity. The mice were divided into five experimental groups (*n* = 10), four receiving different vaccine candidates (LipoAgE6, *c*LipoAgE6, LysMAgE6, and CytAgE6) and one control group with naïve mice (PBS). Each cage contained one mouse from each experimental group (*n* = 5/cage).

### Immunization Protocol

**Figure 2:**
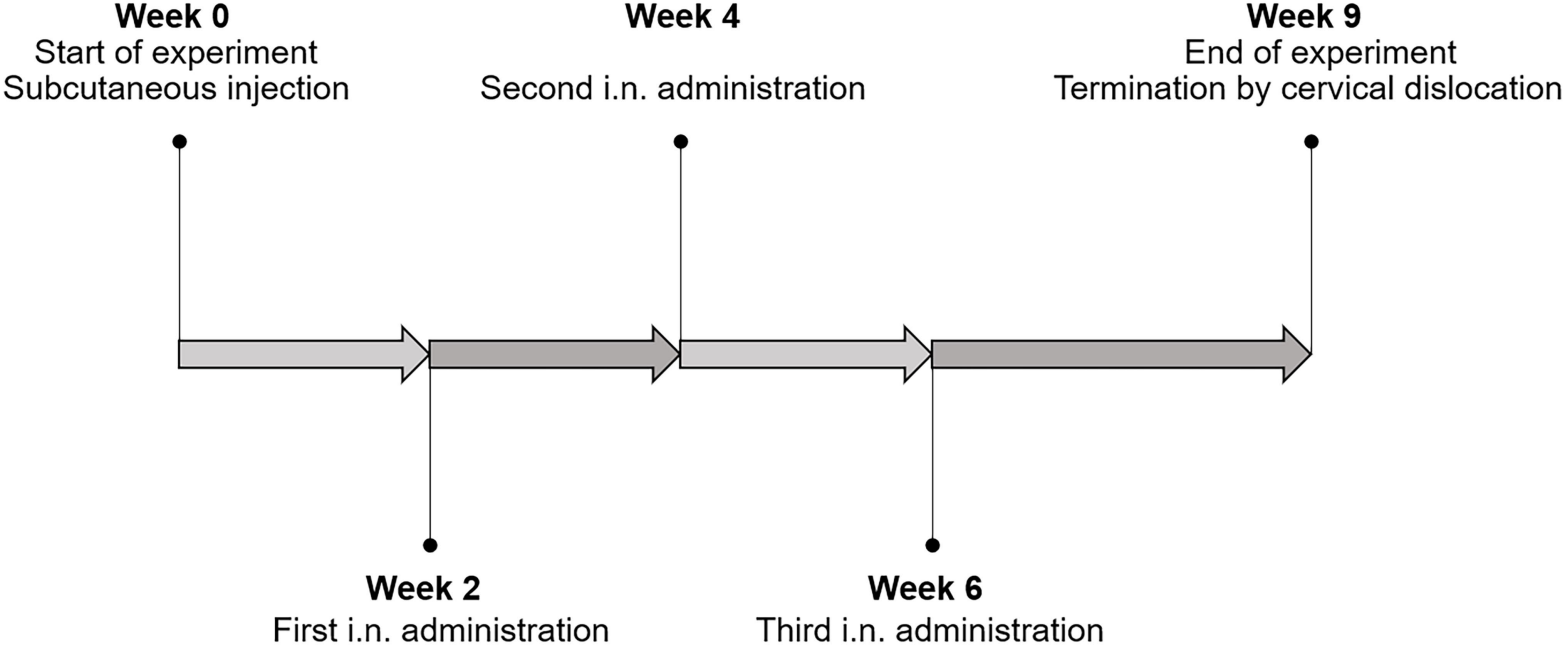
The immunization schedule. All mice were primed with a subcutaneous injection at the start of the experiment (week 0), followed by three intranasal administrations at week 2, 4, and 6. The mice were terminated 3 weeks after the last immunization (week 9).

In total, each mouse was immunized four times (Figure 2). Each immunization dose consisted of ~1×10^9^ UV-inactivated bacteria grown and harvested as described above. The bacterial cultures were counted in a Bürker chamber (Paul Marienfeld GmbH & Co. KG, Lauda-Königshofen, Germany) before harvesting. Harvested bacterial cells were diluted to a density of 6 × 10^8^ bacteria×ml^−1^ in PBS and inactivated by UVB irradiation for 10 min. The UV-inactivated bacteria were centrifuged (5,000 × g for 10 min at 4°C) and the pellets were stored at −80°C until use. To verify successful inactivation of the bacteria, 10 μl of the inactivated bacterial suspensions were transferred to fresh MRS media and incubated overnight. Overnight growth was not observed.

The first immunization was given as a subcutaneous injection (100 μl) at week 0 of the experiment, while the following three vaccinations were given intranasally (25 μl/nostril) at weeks 2, 4, and 6 (Figure 2). The UV-inactivated bacteria were resuspended in PBS supplemented with 20 μg of the T-cell adjuvant poly(I:C; 10 mg/ml; SiSgma-Aldrich) per 1 × 10^9^ UV-inactivated bacteria, shortly before immunization. The mice were placed in an isoflurane chamber prior to immunization. The naïve mice received PBS and the poly(I:C) adjuvant only. The mice were terminated 3 weeks after the last immunization by cervical dislocation (Figure 2). The order of immunization and termination were randomized. The mice were given ordinal numbers in a randomized order on the termination day for the purpose of keeping the samples blinded through the subsequent analyses.

### Sampling of Lung Washes

Lungs from terminated, intact mice were washed with 1 ml sterile PBS and the washes were collected and stored at −80°C until analysis. The samples were centrifuged after thawing and transferred to a new microcentrifuge tube for removal of red blood cell debris.

### Isolation of Total Lung Cells

After sampling of the lung washes from intact mice, the lungs were collected and washed with PBS in a petri dish to remove excess blood. Subsequently, the lung was transferred to a C-tube (Miltenyi) containing 4.5 ml RPMI medium, and kept on ice. Immediately before dissociation, 500 μl of a 10X Collagenase IV (1,250 U/ml) /DNase I (10 MU) enzyme cocktail was added. The lung was dissociated using gentleMACS^™^ Dissociator. Subsequently, the dissociated lung was incubated at 37°C for 1 h under constant horizontal shaking (300 rpm). Following this incubation, the lung was further disrupted using m_lung_02 settings. For total lung cell isolation, the dissociated lung was filtrated through a 70 μm cell strainer. Following centrifugation (300 g for 5 min at 4°C), the filtrate was resuspended in 10 ml 1X RBC lysis buffer (Thermo Fisher Scientific) and incubated on ice for 5 min after which the cell suspension was neutralized by the addition of 15 ml RPMI. After centrifugation (300 g for 5 min at 4°C), the lung cells were resuspended in complete RPMI medium. The cells were stained with 5 μM carboxyfluorescein succinimidyl ester (CFSE) for 20 min at room temperature in the dark, before the stain was removed, and cells were resuspended in complete RPMI and seeded.

### Isolation of Splenocytes

Spleens were collected from terminated mice, transferred to C-tubes (Miltenyi) containing 2.7 ml RPMI medium (Sigma-Aldrich) and kept on ice. Immediately before dissociation, 300 μl of a 10X Collagenase IV (1,250 U/ml; Thermo Fisher Scientific) /DNase I (10 MU; Sigma-Aldrich) enzyme cocktail was added. The C-tubes containing the spleens were placed in a gentleMACS^™^ Dissociator (Miltenyi) for tissue dissociation. Subsequently, the dissociated spleens were incubated at 37°C for 10 min under constant horizontal shaking (300 rpm). After the incubation, the spleen was further disrupted using program m_spleen_03 and kept on ice until splenocyte isolation. The dissociated spleen was centrifuged for 2 min at 300 g and filtrated through a 70 μm cell strainer (Corning, NY) and centrifuged again (300 g for 5 min at 4°C). The filtrate was then incubated with 1X RBC lysis buffer (Thermo Fisher Scientific) on ice for 5 min. The suspension was neutralized by addition of 15 ml RPMI. After centrifugation (300 g for 5 min 4°C), the splenocytes were resuspended in complete RPMI medium. The cells were stained with 5 μM CFSE for 20 min at room temperature in the dark. Then, excess stain was removed, and the cells were resuspended in complete RPMI and seeded.

### Stimulation of Splenocytes and Total Lung Cells

Freshly isolated and CFSE-stained splenocytes and total lung cells were seeded in individual 96-well plates (in duplicate), and stimulated with 5 μg/ml Ag85B, 1 μg/ml ESAT-6, or 1 × 10^5^ Dynabeads^®^ Mouse T-Activator CD3/CD28 (Thermo Fisher Scientific), or not stimulated (negative control). Cells were stimulated with a lower concentration of ESAT-6 due to previously observed cytotoxicity at higher concentrations (Kuczkowska et al., 2019b). Cells were maintained in complete RPMI medium in a humidified incubator at 37°C and 5% CO_2_ for 5 days, after which analysis of antigen-specific CD4^+^ T-cell proliferation (CFSE content), IFN-γ and IL-17A production were assessed as described below.

### Analysis of IgA in Lung Washes

Enzyme-linked immunosorbent assay (ELISA) were performed to determine titers of antigen-specific IgA in the lung wash samples. Microtiter plates were coated with 5 μg/ml Ag85B or ESAT-6 (Lionex GmbH, Braunschweig, Germany) and incubated overnight at 4°C. The plates were then washed with the washing buffer (0.1% Tween-20 in PBS) and blocked with PBS/1% BSA. The plates were incubated with the blocking solution for 1 h at 37°C. The plates were washed three times with the HydroSpeed^™^ plate washer (TECAN, Männedorf, Switzerland), after which the samples, in two-fold serial dilutions, were applied to the coated plates, followed by incubation for 1 h at 37°C. After incubation, the plates were washed three times with the washing buffer, followed by exposure to HRP-conjugated anti-mouse IgA (1,1,000 dilution; Sigma-Aldrich) in 1% BSA 0.05% Tween-20 in PBS for 1 h at 37°C. The plates were washed five times with PBS/0.1% Tween-20 before addition of the eBioscience^™^ TMB Solution (1X; Thermo Fisher Scientific) for color development. The reaction was stopped after 15 min with the ELISA Stop Solution (Thermo Fisher Scientific), and the OD_450_ was measured using a Multiskan^™^ FC Microplate Photometer (Thermo Fisher Scientific). PBS samples were included on all plates and used to set the threshold for calculations of the titer in the lung wash samples.

### T-Cell Proliferation

Flow cytometry was performed on the CFSE-stained stimulated splenocytes and total lung cells to determine antigen-specific T-cell proliferation. After 5 days of stimulation, the cells were harvested by centrifugation for 1 min at 800 × g at 4°C. The supernatant was transferred to a new 96-well plate and frozen at −80°C for later analysis of IFN-γ and IL-17A. The cells were resuspended in 200 μl PBS and transferred to a 96 U-well plate. After centrifugation, cells were stained using the LIVE/DEAD^™^ Fixable Violet Dead Cell Stain Kit diluted 1:100 (Thermo Fisher Scientific), and incubated at room temperature for 30 min, protected from light. The LIVE/DEAD staining was by mistake added at too high concentration, thus inadvertently staining live cells; nevertheless, dead cells could be distinctly separated from live cells as LIVE/DEAD Violet^high^. Fluorescent-minus-one controls and single stained controls were used to exclude any spillover effects, including from the LIVE/DEAD Violet channel. Next, the samples were blocked with FC block (BioLegend, San Diego, CA), 1:100 diluted, and incubated for 10 min at room temperature. After centrifugation, samples were stained with an antibody cocktail containing CD4-PE, CD45-PerCP/Cy5, and CD3-APC/Cya7 antibodies produced in rat, diluted 1:50 (all from BioLegend). The samples were incubated for 20 min at 4°C. The cells were then washed three times with PBS/1% BSA, before fixation of samples with 100 μl IC Fixation buffer (Thermo Fisher Scientific) for 10 min at 4°C, followed by addition of PBS/1% BSA. The plates were sealed and stored at 4°C in the dark until the flow cytometry analysis was performed with a Gallios flow cytometer (Beckman Coulter, Brea, CA). Data were analyzed using Kaluza software (Beckman Coulter).

### Cytokine Analysis

Antigen-induced IFN-γ and IL17-A secretion in total lung cells and splenocytes were quantified by ELISA using the IFN-γ Mouse Uncoated ELISA Kit and the IL-17A (homodimer) Mouse Uncoated ELISA Kit (both from Thermo Fisher Scientific). The cytokine analyses were performed following the manufacturer’s instructions using Costar 3590 plates (Corning). Color development was stopped with ELISA Stop Solution (Thermo Fisher Scientific), and the plates were read using a Multiskan^™^ FC Microplate Photometer at OD_450_.

### Statistical Tests

Data from all analyses were Log10-transformed to meet the ANOVA assumptions prior to statistical testing (data not shown). Statistical significance was determined by one-way analysis of variance (ANOVA) followed by Games-Howell post-hoc test using jamovi version 1.6. (Sydney, Australia). Results are presented as means ± the standard errors of the means (SEM).

## Results

### Characterization of AgE6 Production in *Lactiplantibacillus plantarum*

The mycobacterial Ag85B-ESAT-6 (AgE6) hybrid antigen produced by *L. plantarum* was either directed to the cytoplasma (CytAgE6) or to the surface (Figure 1). In the strains LipoAgE6 and *c*LipoAgE6, the antigen is covalently bound to the cell membrane by an N-terminal lipoprotein anchor derived from the *L. plantarum* protein Lp_1261 (Fredriksen et al., 2012). In the LysMAgE6 strain, the LysM anchoring motif derived from Lp_3014 permits non-covalent attachment to the peptidoglycan layer.

**Figure 3:**
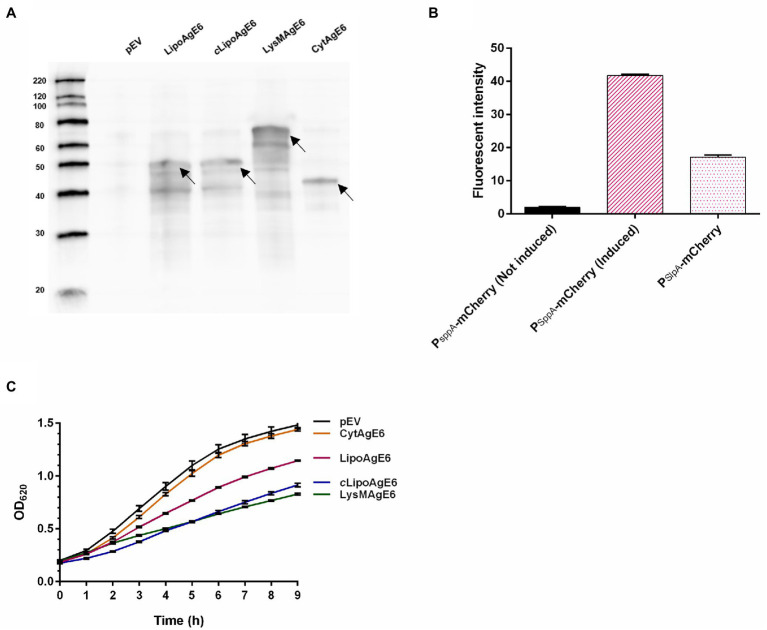
Characterization of the vaccine candidates. **(A)** Western blot analysis of crude protein extracts of the Ag85B-ESAT6-DC (AgE6) expression strains. The volume of loaded samples was adjusted based on the OD_600_ upon harvesting, meaning that approximately equal amount of protein was loaded. Lanes: left lane, molecular marker (masses indicated in kDa); pEV, negative control; LipoAgE6 (50 kDa), *c*LipoAgE6 (50 kDa; constitutive promoter), LysMAgE6 (64 kDa), and CytAgE6 (44 kDa). The arrows point to the bands at the correct sizes. **(B)** Analysis of promoter strength using the fluorescent protein mCherry as a reporter gene. The fluorescence was measured 3 h after induction or 5 h of growth for P*_sppA_*-mCherry and P*_slpA_*-mCherry, respectively. P*_sppA_* is the inducible promoter used in most constructs and P*_slpA_* is the constitutive promoter used in *c*LipoAgE6. **(C)** Growth of the recombinant strains. The cultures were prepared as described in materials and methods. Induced cultures were transferred to a 96-well plate in eight technical replicates after induction (time = 0). The OD_620_ was measured continuously, every 15 min, for 9 h. Every fourth datapoint is shown as the mean of eight technical replicates ± standard deviation to emphasize that the growth curve was measured continuously. The data presented in **A**, **B**, and **C** are from one representative experiment, but all analyses have been performed three times, showing similar results.

Western blot analysis of the crude protein extract showed antigen production by all strains (Figure 3A). AgE6 was detected at the correct size in all recombinant strains, but not in the control strain (pEV). The presence of additional bands lower than the expected size may be due to protein degradation, as commonly observed in similar experiments (Dieye et al., 2003; Le Loir et al., 2005; Kuczkowska et al., 2019b; Mathiesen et al., 2020). Interestingly, this phenomenon was observed to a lesser extent for CytAgE6 with intracellular antigen. The signal intensities detected for the four antigen-producing strains were similar indicating that protein production levels were similar. Construction of P*_sppA_*-mCherry and P*_slpA_*-mCherry enabled comparison of promoter strength between the inducible P*_sppA_* and constitutive P*_slpA_* promoters. Figure 3B reveals a considerable difference in the fluorescent light emitted from the two mCherry strains 3 h after induction, and show that P*_sppA_* is the stronger promoter. Accordingly, the western blot (Figure 3A) showed somewhat fainter bands for *c*LipoAgE6 compared to LipoAgE6.

The production and secretion of heterologous proteins may exert considerable stress on the producer (Bolhuis et al., 1999; Mathiesen et al., 2020; Oh et al., 2021). Thus, the growth of antigen-producing *L. plantarum* was followed for 9 h post-induction (Figure 3C). The growth analysis revealed that CytAgE6 had a similar growth rate to pEV, while growth of the strains with surface-anchored AgE6 was impaired (Figure 3C). The impaired growth may indicate stress associated with translocation of the antigen. It is worth noting that, of LipoAgE6 and *c*LipoAgE6, the strain with the strongest, but inducible, promoter, LipoAgE6, showed better growth.

**Figure 4:**
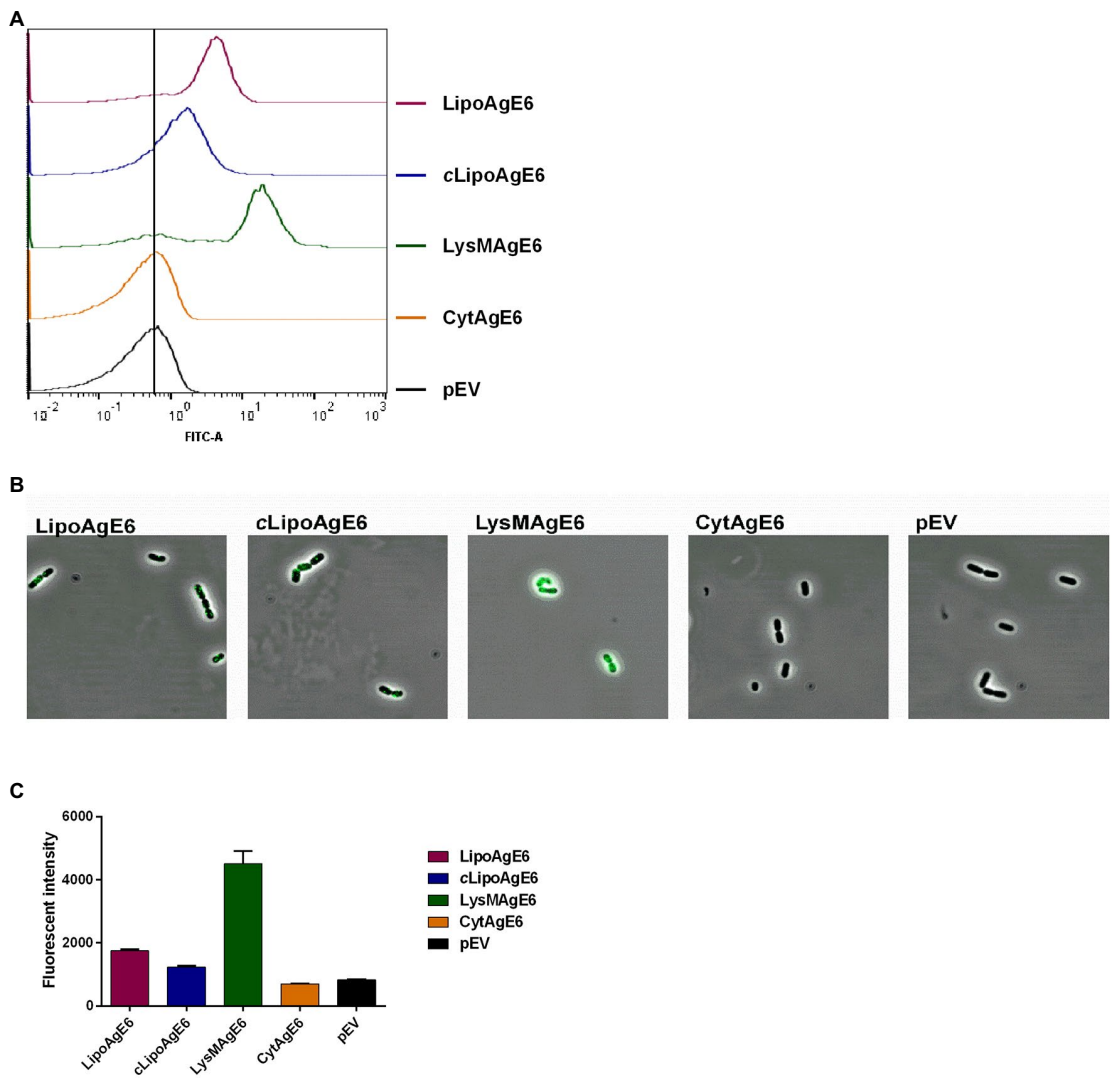
Analysis of surface display of Ag85B-ESAT6-DC (AgE6) by flow cytometry **(A)** and indirect immunofluorescence microscopy **(B)**. Panel **C** shows a bar chart for comparison of fluorescent intensity of the FITC signal for the various strains as derived from the image analysis. The number of bacteria analyzed for evaluation of FITC signal were 215 for LipoAgE6, 93 for cLipoAgE6, 120 for LysMAgE6, 50 for CytAgE6, and 141 for pEV. The data are from one representative experiment out of at least three experiments. The error bars show the standard deviation.

Flow cytometry was performed on intact bacteria to examine surface localization of AgE6 (Figure 4A). As expected, the negative control (pEV) exhibited no fluorescent signal and neither did CytAgE6. The three strains with surface-displayed antigen, LipoAgE6, *c*LipoAgE6, and LysMAgE6, showed a substantial increase in fluorescent signal compared to pEV. The fluorescent signal of *c*LipoAgE6 was slightly weaker than LipoAgE6, which is in accordance with the fainter bands in the western blot and the results of promoter analysis (Figures 3A,B). LysMAgE6, which anchors the antigen non-covalently to the peptidoglycan layer, exhibited the strongest fluorescent signal of all five analyzed strains. The results from flow cytometry were confirmed with fluorescence microscopy (Figure 4B). A bar graph showing the fluorescent intensity from the microscopy analysis is shown Figure 4C.

### LipoAgE6, LysMAgE6, and CytAgE6 Induce Antigen-Specific IgA

**Figure 5:**
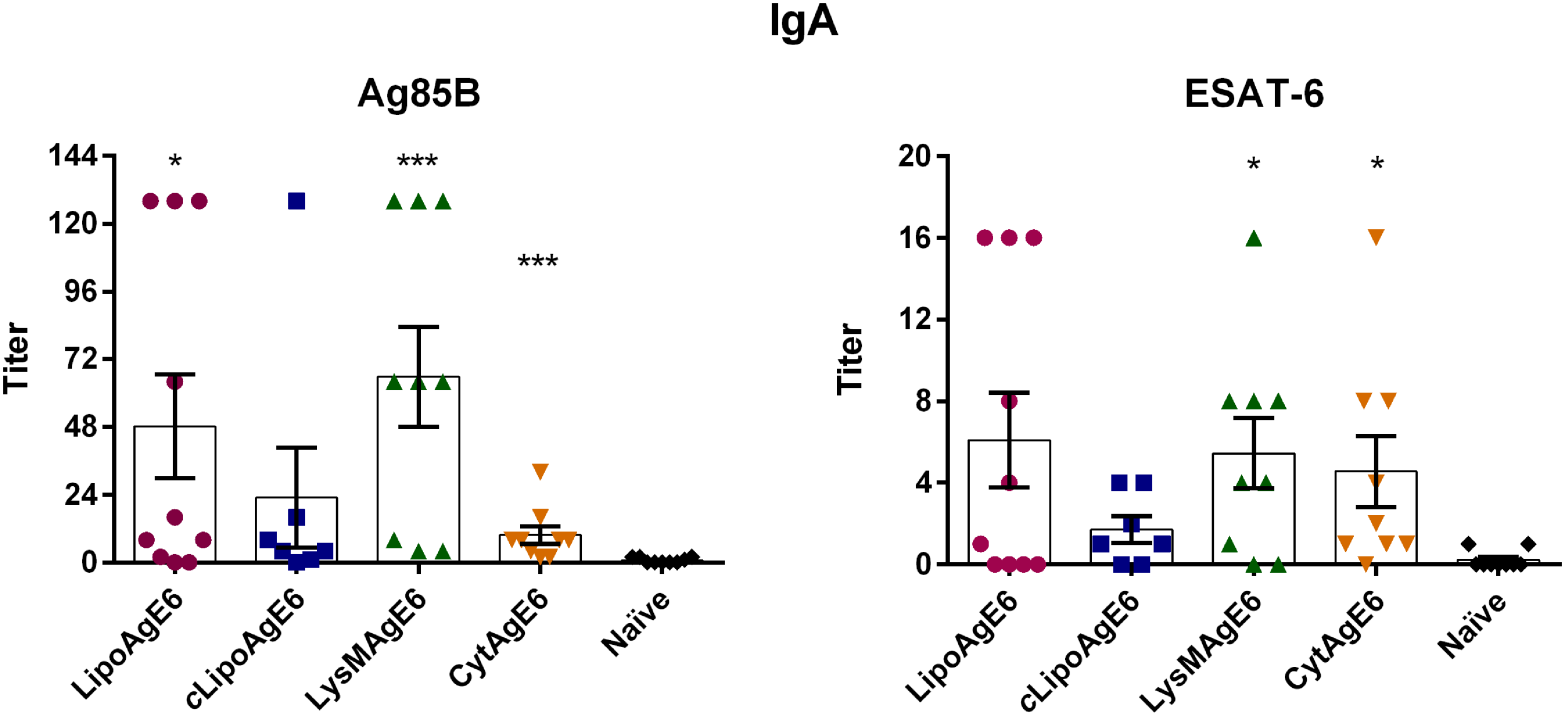
Evaluation of the ability of the four vaccine candidates to induce humoral immunity by analysis of antigen-specific IgA secretion in lung washes. Serial dilutions of lung wash samples (two-fold) were analyzed in an enzyme-linked immunoassay for determination of end-point titers of IgA specific to Ag85B or ESAT-6. Results are presented as a mean ± SEM (*n* = 7–10). Statistics were performed on log-transformed data (data not shown) to meet the ANOVA assumptions. Some samples gave a reported value of 0; therefore, a pseudocount of 1 was added to all samples to avoid irrational values upon log10 transformation. Statistically significant differences relative to naïve mice were determined using one-way ANOVA with Games‑Howell *post-hoc* test. ^*^p < 0.05 and ^***^p < 0.001.

Production of IgA is considered the first line of defense at mucosal surfaces. Thus, the vaccine candidate strains’ ability to stimulate induction of humoral response was assessed by analysis of Ag85B and ESAT-6 specific secreted IgA in lung washes from immunized mice (Figure 5). Interestingly, both Ag85B and ESAT-6 specific IgA titers were significantly increased in lung washes from mice immunized with CytAgE6 and LysMAgE6 compared to naïve mice (Figure 5). The strains with lipoprotein-anchored antigen (*c*LipoAgE6 and LipoAgE6) also showed elevated IgA titers, which, however, was only statistically significant for the Ag85B specific IgA titer in lung washes from LipoAgE6, due to high dispersion among the samples.

### LipoAgE6, *c*LipoAgE6, and LysMAgE6 Leads to Antigen-Specific T-Cell Proliferation in Total Lung Cells

We isolated splenocytes and total lung cells (TLC) to evaluate the cellular responses evoked by the recombinant strains after intranasal immunization of mice. In a previous experiment, LipoAgE6 did not show antigen-specific T-cell proliferation in the spleen (Kuczkowska et al., 2019a,b). In this previous study, the corresponding cellular immune response in the lung was not explored. The present study included three never-before-tested candidates (*c*LipoAgE6, LysMAgE6, and CytAgE6) in addition to the previously tested LipoAgE6. All analyses of cellular responses were performed using both splenocytes and TLC for all candidates.

**Figure 6:**
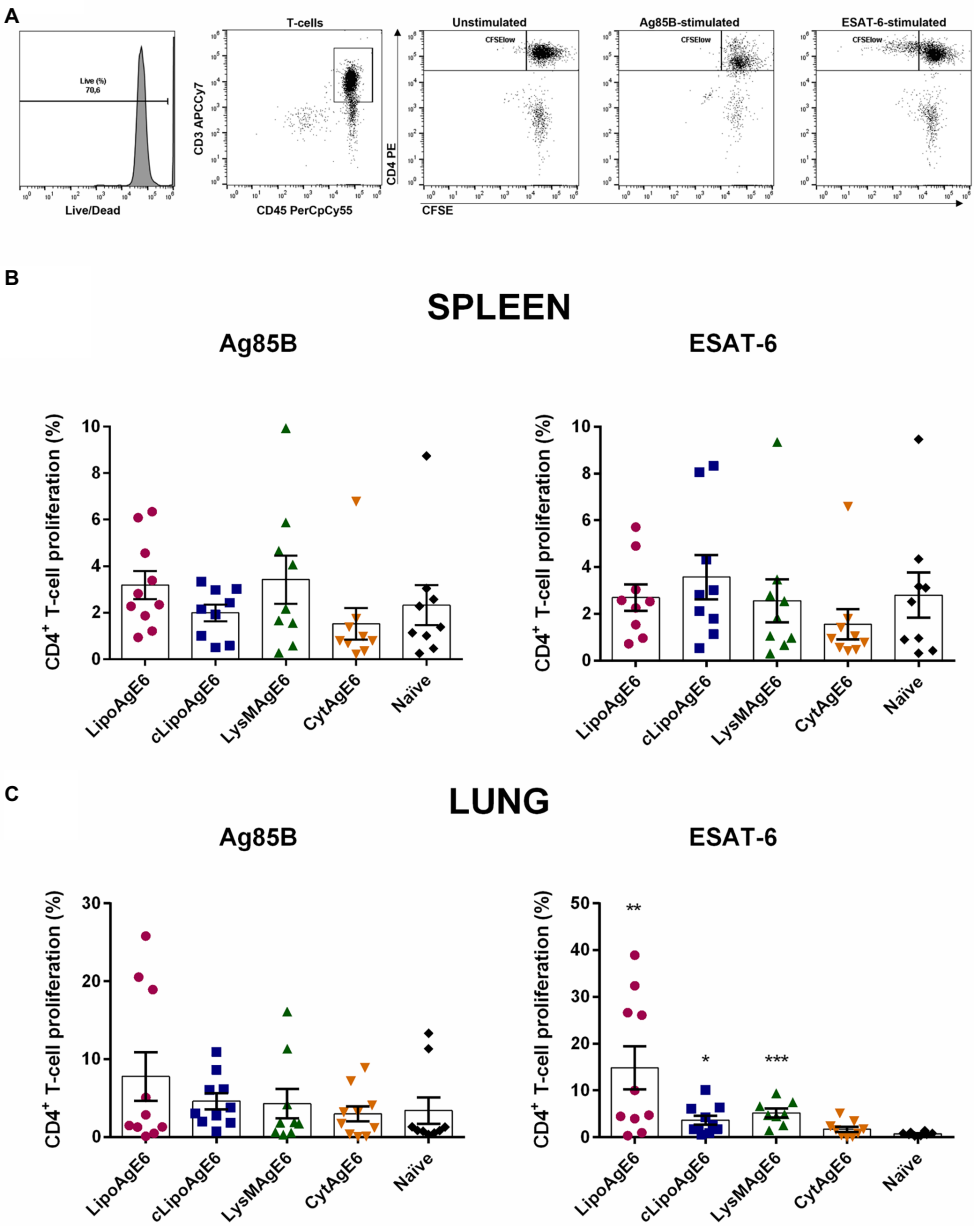
Antigen-specific T-cell proliferation. **(A)** CD4^+^ T-cell proliferation was determined by analyzing the carboxyfluorescein succinimidyl ester (CFSE) fluorescence intensity after stimulation with Ag85B and ESAT-6, using unstimulated cells as a threshold for non-specific proliferation. Specific CD4^+^ T-cell proliferation of splenocytes and total lung cells was determined using a single cell ➔ mononuclear cell ➔ live ➔ CD3^+^ ➔ CD4^+^ ➔ CFSE^high/low^ gating strategy. Panels **B**,**C** show flow cytometry-based quantification of CD4^+^ T-cell proliferation for CFSE-stained splenocytes **(B)** and total lung cells (TLC; **C**) from immunized mice that were stimulated with Ag85B or ESAT-6 for 5 days. Results are presented as mean ± SEM of the samples (*n* = 8–10). Statistics were performed on log-transformed data (data not shown) to meet the ANOVA assumptions. Statistically significant differences relative to naïve mice were determined using one-way ANOVA with Games-Howell post-hoc test. ^*^p < 0.05, ^**^p < 0.01, and ^***^p < 0.001.

After 5 days of antigen stimulation, the CFSE-stained cells were prepared for flow cytometry, subjected to LIVE/DEAD^™^ staining, followed by immunostaining with a mixture of specific antibodies (CD4-PE, CD45-PerCP/Cy5, and CD3-APC/Cya7). The percentage of antigen-specific proliferating CD4^+^ cells were determined based on CFSE intensity. Unstimulated CFSE-stained cells were used to set a proliferation threshold (CFSE^high^). Cell populations with a CFSE fluorescent signal below this threshold were denoted as CFSE^low^ cells and considered to be antigen-specific proliferating cells (Figure 6A). Stimulation with the positive control (Dynabeads^®^ Mouse T-Activator CD3/CD28) validated the method and showed strong activation of T-cell proliferation, detected as a large CFSE^low^ population outside the CFSE^high^ gate (not shown). Compared to the group of naïve mice, significant CD4^+^ T-cell proliferation was observed in the ESAT-6-stimulated TLCs from mice immunized with either of the AgE6 surface-displaying strains (LipoAgE6, *c*LipoAgE6, or LysMAgE6; Figure 6B). Neither the splenocyte populations nor any of the Ag85B-stimulated TLC populations exhibited significant CD4^+^ T-cell proliferation (Figures 6B,C).

### LipoAgE6, *c*LipoAgE6, and LysMAgE6 Induce Antigen-Specific IFN-γ Secretion in Splenocytes and TLC

**Figure 7:**
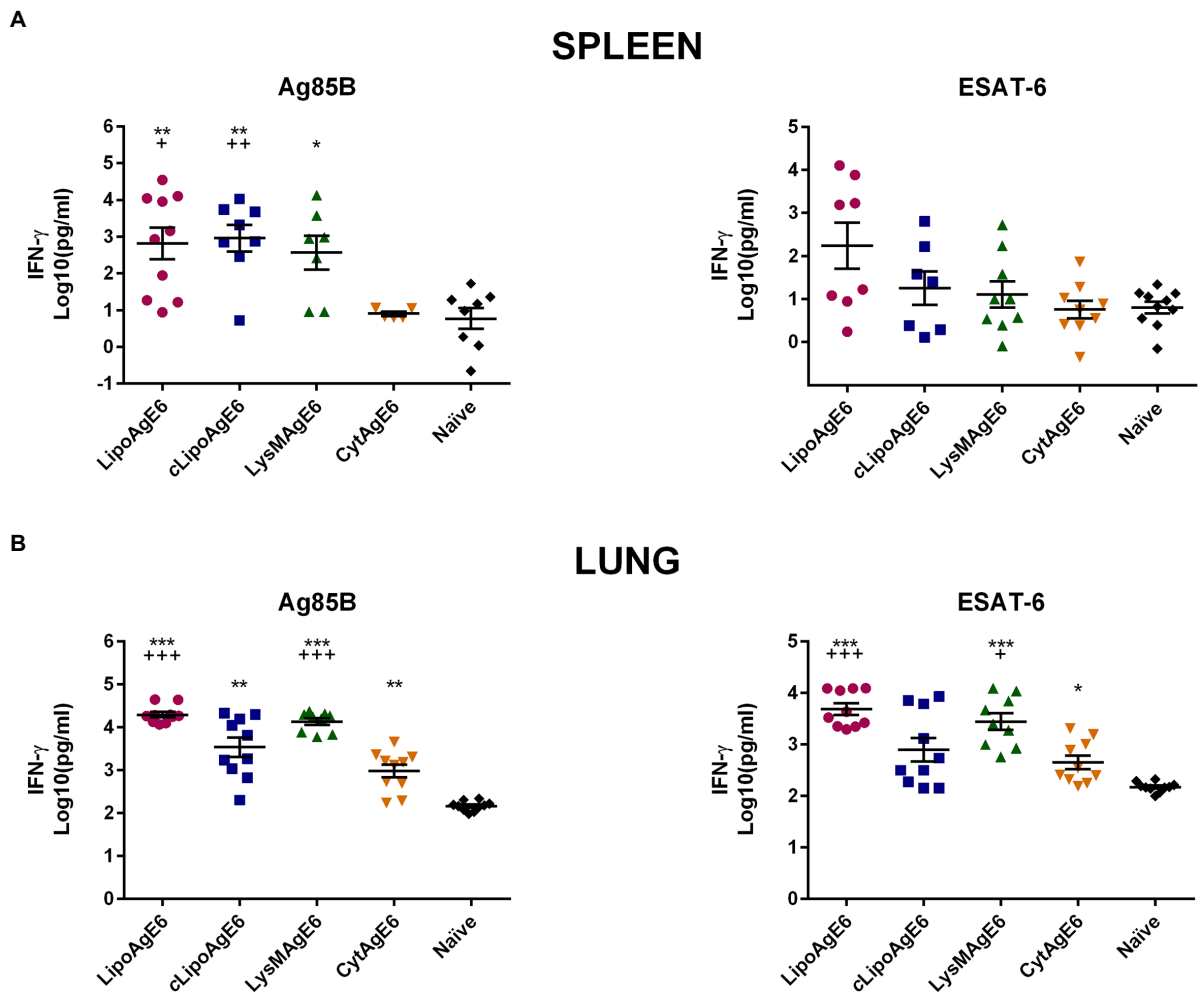
The concentration of secreted IFN-γ in supernatants of splenocytes and total lung cells (TLC) induced by the recall antigens Ag85B or ESAT-6. Splenocytes **(A)** and TLC **(B)** from immunized mice were stimulated with Ag85B or ESAT-6 for 5 days, after which the levels of secreted IFN-γ were quantified by ELISA. Results are presented as a mean ± SEM of samples (*n* = 5–10). The data were Log10-transformed to meet the ANOVA assumptions. Statistically significant differences were determined using one-way ANOVA with Games-Howell post-hoc test. Significant differences between either of the candidates and naïve mice or CytAgE6 are marked with ^*^ and + respectively. ^*/+^*p* < 0.05, ^**/++^*p* < 0.01 and ^***/+++^*p* < 0.001.

Th1 cell production of the cytokine IFN-γ is important to impede the growth of *M. tuberculosis* inside macrophages (Bhatt and Salgame, 2007). Therefore, we analyzed the amount of secreted IFN-γ in cell supernatants from both splenocytes and TLCs stimulated with Ag85B or ESAT-6 (Figure 7). Ag85B-stimulated splenocytes from LipoAgE6, *c*LipoAgE6 and LysMAgE6 immunized mice had significantly higher concentrations of IFN-γ in the cell supernatants compared to naïve mice (Figure 7A). Notably, LipoAgE6 and *c*LipoAgE6 exhibited significantly higher IFN-γ levels also compared to CytAgE6. The IFN-γ level for LysMAgE6 was considerably higher than for CytAgE6, although not significantly. The concentrations of secreted IFN-γ in the supernatants of ESAT-6-stimulated splenocytes were not significantly elevated for neither of the candidates. However, IFN-γ concentrations seemed slightly elevated for cells from LipoAgE6 immunized mice.

Interestingly, in contrast to the spleen, for lung cells increased IFN-γ levels were observed for cells from CytAgE6 immunized mice after stimulation with both Ag85B and ESAT-6 (Figure 7). Nonetheless, IFN-γ levels for mice immunized with LipoAgE6 and LysMAgE6 were significantly increased compared to CytAgE6. Thus, the overall tendency is that the vaccine candidates with surface-anchoring induced higher levels of secreted IFN-γ than CytAgE6, in both lung and spleen.

### LipoAgE6, *c*LipoAgE6, and LysMAgE6 Induce Antigen-Specific IL-17A Secretion in Splenocytes and Total Lung Cells

**Figure 8:**
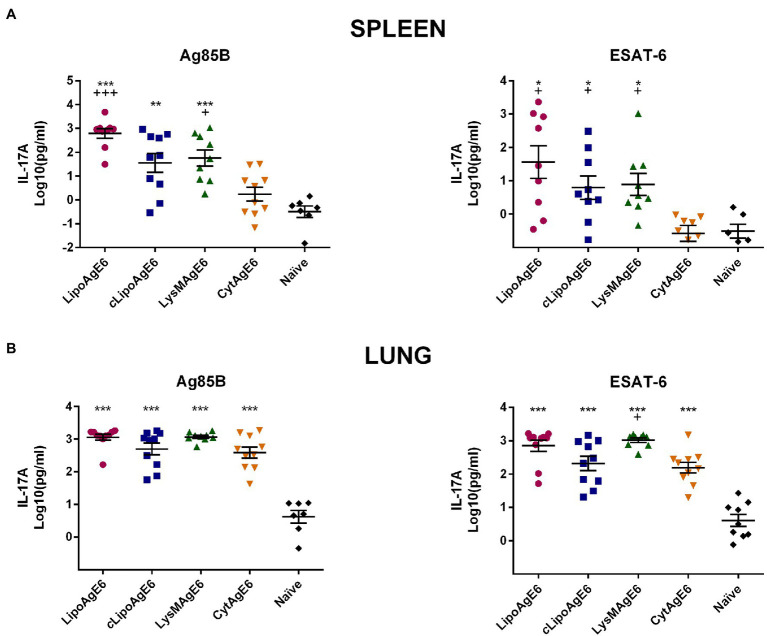
The concentration of secreted IL-17A in supernatants of splenocytes and total lung cells (TLC) induced by recall antigens Ag85B or ESAT-6. Splenocytes **(A)** and TLC **(B)** from immunized mice were stimulated with Ag85B or ESAT-6 for 5 days, after which levels of secreted IL-17A were quantified by ELISA. Results are presented as a mean ± SEM of samples (*n* = 6–10). Statistically significant differences were determined using one-way ANOVA with Games-Howell post-hoc test. Significant differences between either of the candidates and naïve mice or CytAgE6 are marked with ^*^ and +, respectively. ^*/+^*p* < 0.05, ^**/++^*p* < 0.01, and ^***/+++^*p* < 0.001.

Furthermore, we investigated the potential of splenocytes and TLCs from immunized mice to produce and secrete IL-17A upon restimulation with Ag85B and ESAT-6 (Figure 8). The Th17 cytokine IL-17 is assumed to promote immunity during *M. tuberculosis* infection (Counoupas and Triccas, 2019). The analysis showed that IL-17A secretion by splenocytes stimulated with Ag85B and ESAT-6 was significantly higher for cells from mice immunized with surfaced displayed antigen, compared to cells from naïve mice and mice immunized with intracellular TB antigen (Figure 8A).

Secretion of IL-17A by TLC was significantly increased for all four vaccine candidates compared to naïve mice, for both antigen stimulants. There was little variation in the average IL-17A levels in the supernatant of TLCs isolated from either of the four immunized groups, especially for the Ag85B stimulated samples. Notably, the IL-17A concentration in the supernatants of TLCs from CytAgE6 immunized cells was clearly higher than the concentrations seen with splenocytes. Taken together the data of Figure 7 show that, in general, secretion of IFN-γ and IL-17A was increased in splenocytes and TLC from mice immunized with either of the surface-displaying vaccine candidates (LipoAgE6, *c*LipoAgE6, LysMAgE6) whereas immunization with CytAgE6 only led to increased cytokine levels in the lung.

## Discussion

The use of *L. plantarum* comprises a promising strategy in the development of mucosal vaccine delivery systems. Previous studies have shown that antigen display on the surface of *L. plantarum* successfully induces specific immune responses (Fredriksen et al., 2012; Kuczkowska et al., 2017b, 2019a). However, these studies addressed the impact of antigen surface display to a limited extent only. Therefore, the current study was aimed at unraveling the significance of antigen exposure for obtaining specific humoral and cellular immune responses. In addition, we compared the immune responses generated by strains harboring an inducible or a constitutive expression system for a cell membrane-anchored antigen.

Constitutive expression of a target antigen is a more straightforward, faster, and more economical approach compared to inducible expression. The strong inducible promoter P*_sppA_* (Brurberg et al., 1997; Sørvig et al., 2005) that controls the transcription of AgE6 in the inducible strains (LipoAgE6, LysMAgE6, CytAgE6) was compared to the constitutive P*_slpA_* promoter (*c*LipoAgE6). Previous data suggest that, when used in *L. plantarum*, P*_slpA_* and P*_sppA_* have comparable strengths (Nguyen et al., 2019). Only minor differences could be observed between LipoAgE6 and *c*LipoAgE6 in the western blot (Figure 3A). The control experiment with mCherry revealed that P*_sppA_* was distinctly stronger than P*_slpA_* (Figure 3B) and a difference in protein production levels was suggested by the flow cytometry of Figure 4A, showing a stronger signal for LipoAgE6 compared to *c*LipoAgE6. Figure 3C reveals that, despite putatively lower protein production levels, constitutive antigen expression reduced bacterial growth to a larger extent compared to induced expression. These results indicate that constitutive antigen production exerts more stress on the bacteria than inducible expression, and that strains with inducible expression may be the most eligible delivery vectors.

Previous studies have shown that intracellular antigen localization can induce immune responses, that in some cases may be comparable to the responses generated by surface-displayed antigens (Grangette et al., 2002; Reveneau et al., 2002; Oh et al., 2021). Therefore, we constructed a vector that produced AgE6 without a signal sequence or an anchoring motif, to compare intracellular localization to surface display of the antigen. Interestingly, CytAgE6 had a growth rate equal to the control strain with the empty vector, while growth of the strains expressing surface-anchored AgE6 was clearly inhibited (Figure 3C). This shows that secretion stress, rather than antigen production as such, is a stress factor. While the good growth of CytAgE6 is an advantage and while immune responses were detected for this strain, in particular antigen-specific cytokine production by lung cells, overall CytAgE6 was the least effective of the tested antigen-producing strains.

Flow cytometry and immunofluorescence microscopy showed successful antigen surface display (Figures 4A–C). It seems reasonable to assume that the variation in fluorescent intensity observed among the four candidate strains in Figure 4 relates to the degree of AgE6 exposure on the bacterial cell. Anchoring of AgE6 to the membrane, as in LipoAgE6 and *c*LipoAgE6, partly embeds the antigen in the rigid peptidoglycan layer, which could result in the observed weaker fluorescent signal. Conversely, non-covalent LysM-mediated anchoring of AgE6 to the cell wall in LysMAgE6 likely gives a more exposed antigen, which the flow cytometry data indeed seem to indicate. While in LysMAgE6 the antigen is presumably the most accessible for interactions with the immune cells, high exposure may render the antigen more susceptible to *in vivo* degradation, relative to the membrane-anchored antigens.

The four vaccine candidates were first administered parenterally to the mice, followed by three intranasal boosters with two-week intervals (Figure 2). Specific immune responses were analyzed 3 weeks after the last immunization. The production and secretion of IgA is an essential hallmark of the mucosal adaptive immune response, and is essential in combating *M. tuberculosis* infection (Neutra and Kozlowski, 2006). It has previously been shown that the susceptibility to *M. tuberculosis* infection increased in IgA knock out mice, demonstrating the role of IgA during infection (Rodríguez et al., 2005; Tjärnlund et al., 2006). Immunization with all four vaccine candidate strains resulted in increased Ag85B and ESAT-6 specific IgA titers in lung washes compared to naïve mice (Figure 5). Immunization with CytAgE6 generated levels of ESAT-6 specific IgA that were not significantly different from the elevated IgA titers in lung washes from mice immunized with either of the vaccine candidates with surface-anchored AgE6. However, CytAgE6 also gave increased titers of Ag85B specific IgA, which were more pronounced. It is interesting to note that also the levels of antigen-induced cytokine secretion (Figures 7, 8; discussed below) indicated that CytAgE6 induced an immune response in the lungs, and much less so in the spleen.

Thus, the IgA results indicate that both surface exposed and cytoplasmic AgE6 can induce a proper mucosal immune response in the lung. Recently, Oh et al. (2021) demonstrated that active release of cytoplasmic antigen from a live *L. plantarum* delivery vector after oral immunization resulted in antigen-specific immune responses. In the current study, however, the bacteria were UV-inactivated; thus, the immune response we observed with CytAgE6 is likely related to spontaneous lysis of the bacteria and subsequent release of enough antigen to stimulate the immune system.

Immunization with either of the three recombinant strains with surface-displayed antigen led to antigen-specific CD4^+^ T-cell proliferation upon restimulation of TLC with ESAT-6, whereas significant antigen-induced T-cell proliferation was not observed for splenocytes (Figure 6). The findings with TLC indicate successful generation of CD4^+^ tissue resident memory cells upon immunization with recombinant *L. plantarum*, provided that the antigens are surface-displayed, which is encouraging. The lack of antigen-induced proliferation of splenocytes is in accordance with previous observations for LipoAgE6 (Kuczkowska et al., 2019a). Interestingly, a challenge study by Forbes et al. (2008) showed that lung resident Th1 cells, which includes CD4^+^ cells, show a stronger correlation with protection compared to spleen resident Th1 cells.

Production of the pro-inflammatory cytokine IFN-γ by activated CD4^+^ T cells is essential for protection against *M. tuberculosis* because IFN-γ promotes the Th1 cell response and subsequent activation of macrophages (Saito and Nakano, 1996; Lighvani et al., 2001; Counoupas and Triccas, 2019). Analysis of antigen-induced IFN-γ levels (Figure 7) showed increased secretion by both lung and spleen cells from mice immunized with the strains that display AgE6 on their surface, compared to CytAgE6. Similarly, using *L. lactis* for delivery, Bermúdez-Humarán et al. (2004) found that intranasal immunization with cell wall anchored human papilloma virus antigen E7 induced higher levels of antigen-specific IFN-γ secretion than cytoplasmic E7. Figure 7 further shows that IFN-γ secretion was generally higher in TLCs than in splenocytes.

Next, to being promoted by IFN-γ, the desirable Th1 response is supported by Th17 cells through production of IL-17, which plays an essential role in the recruitment of CD4^+^ cells in the lung after challenge with *M. tuberculosis* (Khader et al., 2007). Supporting a beneficial effect on the Th1 response, measurements of antigen-induced secretion of IL-17A (Figure 8) showed results similar to this obtained for IFN-γ. Like for IFN-γ, the IL-17A were more elevated for TLC compared to splenocytes. Similar differences between organs after intranasal immunization have been observed previously (Forbes et al., 2008; Flórido et al., 2018). In the present study, the observed differences may indicate that the systemic immune response was not well boosted by the three intranasal immunizations, compared to the response in the lungs. It is also with noting that CytAgE6 had clear immune stimulatory effects in the lungs but less so in the spleen. This may have to do with cell fate, such as the degree of lysis, which may contribute to organ-dependent differences. The vaccination schedule is a key factor in obtaining the best immune response (Pollard and Bijker, 2021). Thus, it is plausible that the immunization schedule can be further optimized to obtain a higher conversion from mucosal to systemic immune response after intranasal immunization.

In conclusion, we found that all four vaccine candidate strains induced specific humoral and cellular immune responses in the spleen and lung. The immune response detected in the lungs is highly advantageous because the lung is the primary site of *M. tuberculosis* infection. Inclusion of strains LipoAgE6 and *c*LipoAgE6 allowed for comparison between inducible and constitutive antigen expression. We found that inducible expression generated stronger immune responses compared to *c*LipoAgE6, however, the difference was not significant. Furthermore, the novel candidate LysMAgE6 induced immune responses at comparable levels to LipoAgE6, showing that LysM anchoring is a feasible strategy. Further research is needed to analyze exactly which factors determine strain success, taking into account the actual degree of antigen exposure, actual *in vivo* degradation, and the actual amount of protein delivered at immunization. While questions remain, it is clear that in this study, LysM anchoring provided sufficient protection against extracellular degradation. The use of LysM domains is interesting, since this in principle allows a non-GMO approach based on charging natural lactobacilli with LysM containing antigens that are produced in a contained environment. In general, the candidate strains with surface-located antigens induced stronger immune responses than CytAgE6. Thus, for this well-known TB fusion antigen, anchoring to the surface is the most promising delivery strategy.

## Data Availability Statement

The raw data supporting the conclusions of this article will be made available by the authors, without undue reservation.

## Ethics Statement

The animal study was reviewed and approved by Norwegian Animal Research Authority (Mattilsynet, Norway).

## Author Contributions

KW, GM, VE, KK, and HC designed the study. KW, LM, and PB performed the experiments and analyzed the data. KW processed the results and drafted the paper. KW, GM, and VE contributed to analyzing the data and finalizing the manuscript. All authors read and approved the final manuscript.

## Funding

This work was funded by a Ph.D. fellowship from the Norwegian University of Life Sciences to KW.

## Conflict of Interest

The authors declare that the research was conducted in the absence of any commercial or financial relationships that could be construed as a potential conflict of interest.

## Publisher’s Note

All claims expressed in this article are solely those of the authors and do not necessarily represent those of their affiliated organizations, or those of the publisher, the editors and the reviewers. Any product that may be evaluated in this article, or claim that may be made by its manufacturer, is not guaranteed or endorsed by the publisher.
